# Why Should a Genome Be Protected? Ethical, Legal, and Security Challenges in the Protection of Genomic Data

**DOI:** 10.3390/biology15090726

**Published:** 2026-05-02

**Authors:** Marlena Szalata, Mikołaj Danielewski, Karolina Wielgus, Ryszard Słomski

**Affiliations:** 1 Department of Biochemistry and Biotechnology, Poznań University of Life Sciences, 60-632 Poznań, Poland; 2 Independent Researcher, 60-374 Poznań, Poland; 3 Regional Centre of Blood Donation and Blood Treatment in Poznań, 60-354 Poznań, Poland; 4 Institute of Medical Sciences, College of Social and Media Culture in Toruń, 87-100 Toruń, Poland

**Keywords:** genetic material, genome, databases, protection, ethical, legal, security

## Abstract

Why should a genome be protected? Because it contains our most private data! A genome contains an organism’s set of genetic material (DNA and, in viruses, RNA), and it contains all genes and non-coding sequences. The structure of DNA was described by Watson and Crick in 1953, but the first studies were conducted a century earlier by Miescher, who described the structure and chemical composition of the nucleus. The first action aimed at securing the results of genetic research was the creation of databases for the results obtained using genetic fingerprinting technology. The discovery of the sequencing method and the introduction of the polymerase chain reaction laid the foundations for understanding the genome’s function. Automated DNA sequencing proved to be hundreds of times faster than traditional methods, thus reducing the cost and time of genome analyses. Thousands of genomic data points are stored in private and governmental databases. The security of patients’ genomic data must be ensured by protecting it from unauthorized use while, at the same time, enabling research for the sake of public health. The falling prices of genome sequencing and the increasing availability of commercial sequencing for the public could result in ethical problems and undermine the safety of personal information.

## Simple Summary

A genome is an organism’s set of genetic material (DNA and, in viruses, RNA), and it contains all genes and non-coding sequences. It is well known that the structure of DNA was described by Watson and Crick, but the first studies were conducted a century earlier by Miescher, who described the structure and chemical composition of the nucleus. The discovery of the sequencing method and the introduction of the polymerase chain reaction laid the foundation for understanding the genome’s function. Currently, thousands of genomic datasets are used for analyses in drug development, pharmacogenomics, personalized medicine, global health, lifestyle, and wellness in the largest market in North America and the fastest-growing market in the Asia–Pacific region. Patient data obtained through high-throughput studies available online require a proper balance between biosafety oversight and the right to privacy. The falling prices of genome sequencing and the increasing availability of commercial sequencing for the public could result in ethical problems and undermine personal information safety.

## 1. Introduction

A genome is an organism’s set of genetic material (DNA and RNA in viruses), and it contains all genes and non-coding sequences. It contains our most private data, comprising instructions for the structure, development, and functioning of an organism. In eukaryotes, the nuclear genome consists of a haploid set of chromosomes; in humans, it also includes mitochondrial DNA, and in plants, it contains chloroplast DNA. The genome consists of genes (coding for proteins or RNA) and regulatory and non-coding sequences (introns and intergenic DNA segments). The human genome consists of over 3 billion DNA base pairs organized into 23 pairs of chromosomes. The term genome is species-specific (the complete set of information), while genotype refers to the set of genes of a specific individual. Genetic data should be treated as sensitive personal data characterized by permanence, since the genome does not change; a data breach results in the permanent disclosure of information. Another characteristic is traceability, which allows individuals to be identified even based on partial data, e.g., for the purposes of forensic genealogy. Heredity also involves having information about family members: biological parents, siblings, and children. Genetic material and knowledge of the genome are predictive in nature, allowing for the forecasting of future health and creating opportunities to prevent the development of many diseases, but they also open the door to potential misuse. It is therefore necessary to ensure adequate protection while raising public awareness. This study aims to present information regarding the genomic data of individuals, the possibilities for its use, and ensuring its security [1].

## 2. The Beginnings of DNA Research

**Figure 1:**
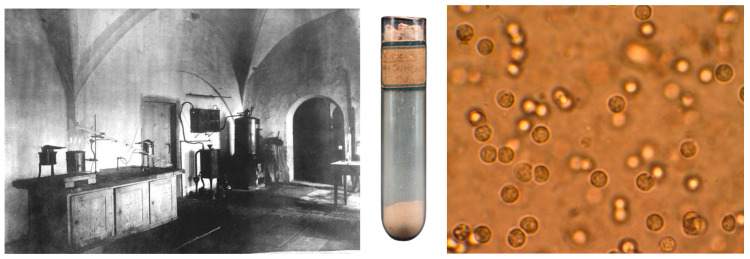
The former kitchen of Tübingen Castle was part of the laboratory where Miescher worked during his stay, where he isolated nuclein. The kitchen, converted into a laboratory, allowed for low-temperature work, which was important for work with nuclein. Unfortunately, the cold in the kitchen contributed to Miescher’s health problems. The glass vial contains nuclein purified by Miescher from salmon. Clusters of leukocytes in pus stained with toluidine. Miescher’s diligence was so intense that he skipped his own wedding to Maria Anna Rüsch, instead working in his laboratory [6]. Reproduced with permission: Tübingen Castle—photo by Paul Sinner, https://commons.wikimedia.org/wiki/File:Schlossk%C3%BCche_Historische_Ansicht_2.jpg (accessed on 29 April 2026); nuclein vial—Museum of the University of Tübingen MUT/V. Marquardt; pus—photo by Bobjgalindo, https://commons.wikimedia.org/wiki/File:Pyuria.JPG (accessed on 29 April 2026).

The structure of DNA was described by James D. Watson and Francis H. C. Crick in 1953, but the first studies were conducted nearly a century earlier by Friedrich Miescher, who, while working in Basel and Tübingen, was interested in the structure and chemical composition of the cell nucleus. Friedrich Miescher isolated nucleic acid in 1869, thus giving rise to the name deoxyribonucleic acid [2]. He digested pus from surgical bandages with pepsin to remove cytoplasmic material. From the undigested residue, Miescher isolated a substance, which, to emphasize its nuclear origin, he named nuclein (Figure 1). Nuclein turned out to be a complex substance composed of a low-molecular-weight basic protein and a second non-protein component possessing the properties of a polybasic acid with significant phosphorus content. While most of the other discoveries and concepts were met with great interest by the scientific community at the time, the discovery of DNA was generally underappreciated [3]. Although uncovering the molecular basis of cellular life had become one of the most fundamental problems of the time, no real significance was credited for his discovery [4]. Miescher later discovered that nuclein is found primarily in chromosomes. Most of the materials that have been preserved come from the efforts of Miescher’s uncle, Wilhelm His, who was both a close friend and important partner for scientific discussions. Together with Miescher’s friends and colleagues, he compiled a collection of his nephew’s work, which was published 2 years after Miescher’s death [5]. These volumes comprise Miescher’s scientific publications, constituting nearly 100 letters by Miescher on various aspects of his research and theories, the manuscripts of his key lectures, and subsequent papers compiled and published posthumously by coworkers based on Miescher’s laboratory notes.

## 3. The Greatest Discovery of the 20th Century

James D. Watson and Francis H. Crick are primarily associated with the discovery of the DNA structure. This international team, working at the Cavendish Laboratory at the University of Cambridge, discovered the structure of DNA, the most important component of every organism. The British–American duo, in collaboration with Rosalind Franklin and Maurice Wilkins, made a discovery considered groundbreaking for the 20th century. Rosalind Franklin and Maurice Wilkins, specialists in chemistry and crystallography, worked at King’s College London. Behind their discoveries’ success, however, lie forgotten figures and the complex histories of many other researchers. James Dewey Watson and Francis Harry Compton Crick worked together for several years. On 28 February 1953, Crick announced, at the Eagle Pub in Cambridge, that he and Watson had discovered the “secret of life” [7]. Of the four discoverers of the structure of DNA, Rosalind Franklin did not receive the Nobel Prize, having died of radiation poisoning [8,9]. Friedrich Miescher and Rosalind Franklin are the forgotten participants in DNA research.

**Figure 2:**
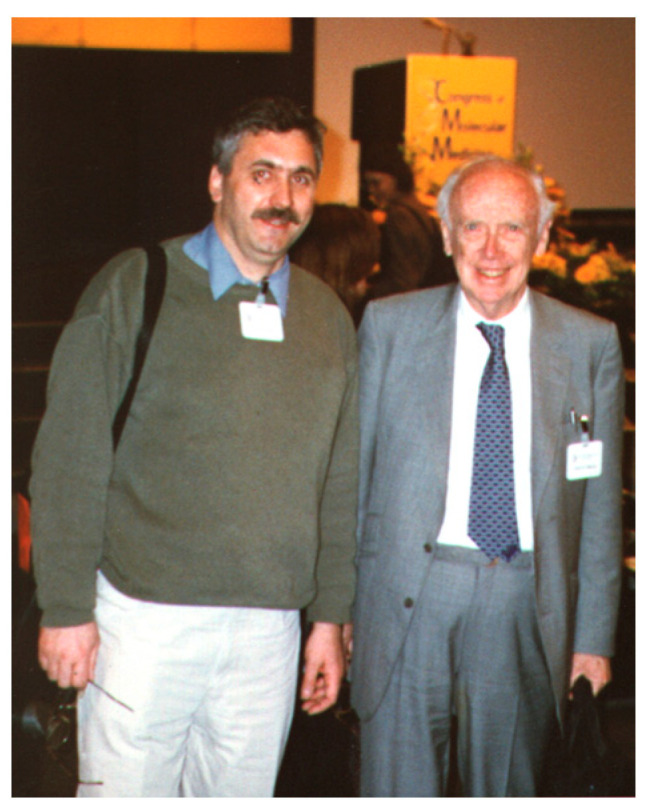
Meeting of one of the authors, Professor Ryszard Słomski, with James D. Watson during the Congress of Molecular Medicine in Berlin on 3 May 1997. The conversation focused on training young researchers in new methods of molecular biology. Thanks to Watson’s support, over 1000 researchers were able to learn modern molecular biology techniques. At the time, Watson was the director of the Cold Spring Harbor Laboratory. Reproduced with permission: photo by Ryszard Słomski.

During the Congress of Molecular Medicine in Berlin on 3 May 1997, Prof. Ryszard Słomski had the opportunity to discuss his controversial lecture, “Genes and Politics,” directly with James D. Watson (Figure 2), during which he also touched on genetics in the Third Reich [10].

## 4. Genetic Fingerprinting

One of the first actions aimed at securing the results of genetic research was the creation of databases for the results obtained using genetic fingerprinting technology. In 1993, the book *DNA Fingerprinting: State of the Science* was published, edited by Sérgio D. J. Pena, Ranjay Chakraborty, Joerg T. Epplen, and Alec J. Jeffreys [11]. It summarized the achievements to date stemming from the 1985 development by Alec Jeffreys and colleagues of *multilocus* molecular probes capable of simultaneously revealing variations in multiple loci in the human genome, known as DNA fingerprinting [12]. Within a few months, the technique enabled the resolution of previously unresolved immigration and paternity disputes, and DNA typing systems are now routinely used in public and commercial forensic laboratories, replacing conventional protein markers as the method of choice for resolving paternity disputes and criminal cases [13]. This method has also entered the natural sciences, as evidenced by a monograph based on the results presented at the Second International Conference on DNA Fingerprinting, held in Belo Horizonte, Brazil, in 1992. One of the authors, Ryszard Słomski, participated in research on the implementation of oligonucleotide probe-based DNA fingerprinting methods for determining paternity in Poland [14,15].

## 5. Understanding the Genome

Sanger sequencing in 1977 [16] and the polymerase chain reaction (PCR) in 1986 [17] are groundbreaking discoveries that have shaped modern molecular biology. As key methods for improving the field, they are not only more efficient but also simpler and more accurate than previously used techniques. Sanger’s method, or the dideoxy method, is a laboratory DNA sequencing technique that determines the nucleotide sequence of a specific DNA molecule, whereas the purpose of PCR is to amplify selected DNA fragments. By combining these strategies, scientists have generated a vast amount of data, the most prominent of which was the Human Genome Project (HGP), completed in 2003. This project sequenced a complete human reference genome for the first time. Because the samples used in the project came from multiple individuals, the resulting genome was considered close to random and, therefore, more representative of a typical human genome than that of a single individual. Because the human genome is approximately 3.2 gigabase pairs (Gbp), a compressed file containing the complete genome can require at least 3 gigabytes of storage. Given the scale of the complete human genome and the need to include all genomic features, intermediate files, and annotations, storage requirements can be problematic [18].

In 2006, Ryszard Słomski participated in a delegation of Polish scientists and businessmen to Houston, Texas. The main event was a series of lectures for invited guests, including Francis Collins, an American physician and geneticist known for groundbreaking discoveries in the field of genetic diseases, who led the Human Genome Project. Our contribution to understanding the genome involves studying the region of human chromosome 9q34, which, among others, contains the *XPMC2H* gene [19].

Our further studies focused on mosaicism, the phenomenon in which a fraction of, rather than all, germline and somatic cells contain a mutation or chromosomal abnormality. It occurs in all genetic disorders in which spontaneous mutations occur and has important clinical consequences for the assessment of patients with localized expression of multisystem disorders, for genetic counseling, and for molecular diagnostic testing, similarly to the case of tuberous sclerosis, an autosomal dominant disorder characterized by the development of unusual tumor-like growths (hamartomas) in multiple organs. The incidence of tuberous sclerosis is 1 in 6000 births, and about two-thirds of cases are sporadic, occurring in the absence of a family history of the disorder. Mutations in one of two genes, *TSC1* and *TSC2*, cause tuberous sclerosis [20].

Genomic data are presented in several specialized file formats, and they are aimed at specific applications. The main formats include the following: FASTA format, presenting plain sequences; FASTQ, a format that extends sequence data with quality data; SAM/BAM/CRAM, used for different alignment maps; BED/GTF, used for storing different genomic annotations; and bedgraph, used to store continuous-valued data (Table 1).

## 6. Reducing the Cost and Time of Genome Analysis

The first automated DNA sequencer filled an entire laboratory. It proved to be hundreds of times faster than traditional sequencing [21]. Leroy “Lee” Edward Hood developed the sequencer in 1986 [22]. Basic data on the human genome were soon obtained. The human genome size, according to the National Center for Biotechnology Information (NCBI, https://www.ncbi.nlm.nih.gov/datasets/genome/GCF_000001405.40/ (accessed on 29 April 2026)), is 3.099 × 10^9^ bp. The number of genes, according to the International Human Genome Sequencing Consortium (IHGSC, https://www.genome.gov/12513430/2004-release-ihgsc-describes-finished-human-sequence (accessed on 29 April 2026)), is 21,787. The number of exons is 231,667. The average number of exons per gene is eight, but there are also genes without exons and those with more than 100 exons. The average exon length is 316 bp, and the intron length is 5747 bp, making exons significantly smaller. The total length of exons is only 1.2% of our genome. Steven Pinker, a professor of psychology at Harvard University, put it this way in “Knowing your own genome”: “But if you want to know if you’re at risk for high cholesterol, get your cholesterol checked; if you want to know if you’re good at math, take a math test. And if you really want to know yourself (and this will be a test of your skills), consider François La Rochefoucauld’s suggestion: “Our enemies’ opinion of us is closer to the truth than our own” [23].

The reduction in DNA testing costs in recent years is primarily due to technological advances (next-generation sequencing, NGS) and increasing market competition. Key factors driving down prices include technological advances and innovations (NGS) that enable massive parallel sequencing, which drastically reduces the cost per sample. The cost of whole-genome sequencing has fallen from billions of dollars two decades ago to several hundred dollars today. The type of biological material used in testing also contributes to lower analysis costs. Cheek swabs are cheaper than microtracer analysis (e.g., hair, blood stains). Using a mail-order kit and self-collecting the sample at home reduces costs compared to a clinic visit. In many countries, e.g., in Poland, the National Health Funds reimburses new genetic tests, including aCGH (array comparative genomic hybridization) and qRT-PCR (quantitative reverse transcription polymerase chain reaction), which allows for significant savings. *BRCA1* and *BRCA2* mutation testing are among the tests reimbursed. Forensic tests, on the other hand, are more expensive because they require strict procedures, witness presence, and proper preservation of the material. In summary, costs are decreasing thanks to NGS technology, and the availability of free tests, particularly in oncology and clinical genetics, has significantly increased in 2025 [18].

Next-generation sequencing (NGS) has significantly accelerated genome discovery because it is high-throughput and even revolutionary. The first generation includes Sanger sequencing, which was the first method of DNA sequencing [16,18,22]. Second-generation sequencing encompasses several technologies: pyrosequencing, terminator chemistry sequencing, and ligation-assisted sequencing. Third-generation sequencing introduces new technologies: fluorescently labeled single-molecule sequencing, real-time single-molecule sequencing, semiconductor sequencing, and nanopore sequencing. The fourth-generation sequencing concept aims to enable the analysis of a cell’s genome directly within the human body. Currently, several dozen genome copies, divided into short fragments (50 bases), are sequenced in parallel. A single study produces DNA sequences measured in gigabases [18,24].

An example of the application of mass sequencing results is the analysis of DNA sequence variants in selected tumor suppressor genes, proto-oncogenes, and genes involved in cell-cycle regulation and inflammatory processes in patients with differentiated thyroid cancer based on high-throughput sequencing techniques [25]. Another example is the search for genetic variants responsible for individual variability in the metabolism of the anesthetics propofol and sevoflurane using high-throughput sequencing technology.

The genome can be studied as a whole, rather than focusing on a single gene. Medical genetics has become part of the broader field of genomic medicine, which seeks to apply large-scale genomic analysis, including the regulation of gene expression, human gene variation, and gene–environment interactions, to improve medical care. Medical genetics focuses not only on the patient but also on the entire family. A comprehensive family history is an important first step in analyzing any disorder, regardless of whether it is known to have a genetic basis.

Osama bin Laden was found and killed by the Central Intelligence Agency (CIA) based on DNA testing. The CIA used a fake vaccination campaign in Abbottabad, Pakistan, in a failed attempt to obtain DNA from Osama bin Laden’s relatives before his killing in 2011. Combining scientific advances in health with intelligence operations is highly controversial and simultaneously results in a reduction in vaccination opportunities and, in this case, the elimination of polio in Northwestern Pakistan, the last major polio reservoir [26].

Knowledge of the full DNA sequence enabled the identification of all human genes—determining the range of variations in these genes across different populations—and outlined how variations in these genes contribute to health and disease. Children with congenital defects undergo routine chromosome analyses and high-resolution genomic testing to detect submicroscopic chromosomal deletions or duplications. A young woman with a family history of breast cancer receives counseling, test interpretation, and support from a counselor specializing in hereditary breast cancer. An obstetrician submits chorionic villus samples from a 38-year-old pregnant woman for cytogenetic testing to determine abnormalities in the fetal chromosome count or structure. The physician combines family and medical history with a genetic study of the young adult patient. An oncologist examines patients to identify genetic changes that may predict a favorable or adverse reaction to a chemotherapy agent. A forensic pathologist utilizes databases of genetic polymorphisms to analyze DNA samples obtained from the personal belongings of victims and living relatives to identify remains. The discovery of an abnormal oncogenic signaling pathway due to a somatic mutation can lead to cancer. It is then possible to develop a specific potent inhibitor of this pathway that effectively treats the cancer [24,25].

In 2008, at the age of 34, Marjolein Kriek from Leiden University became the first woman and the first European to have her genome sequenced. At the time, she was one of five people whose genomes were sequenced, along with James D. Watson, Craig Venter, and two Yoruba men. Each base pair was analyzed seven to eight times on average. A computer printout of a DNA sequence could fill a thousand books of a thousand pages each.

## 7. Biological Databases

In the fall of 2023, the personal data, genetic test results, and geographic locations of 6.9 million 23andMe users, primarily Ashkenazi Jews, Chinese users, and British and German profiles, were exposed on an online forum for cybercriminals [27,28]. Furthermore, the company’s bankruptcy raised concerns about the availability of customer genomic data for scientific use [29,30,31,32]. Where did it come from? If we continue to carelessly share patient data for high-throughput testing, we are not only building databases for commercial, private entities but also, instead of developing our own science, dumping genetic data into a completely unidentified online space, where it can be used for unethical or even bioterrorist purposes. We urgently need a global strategy for genomic data protection based on awareness, control, and accountability [33].

As genome sequencing technology has advanced, the amount of data generated has increased over the last two decades. The wealth of digital sequence information (DSI) has improved our understanding of the genome, expanded research capabilities, and enabled breakthroughs in medicine and industry [34]. The requirements and challenges of storing large DSI files have re-emerged, and combined with the need for easy access for researchers worldwide and the possibility of independent validation, this has led to the creation of many genome databases. Driven by the message that publicly funded data should be accessible, open access has become the dominant form. Another issue that requires addressing and improvement is the growing popularity of commercial genetic testing, which has raised concerns about data misuse and patient privacy [35]. The issue of exploiting overstretched natural resources in least developed countries, the provision of DSI, and the benefits that largely accrue to the most developed countries were raised at the United Nations Conference on Biological Diversity (COP15) in 2022. The solution is financial compensation for data, which could help protect biodiversity. To familiarize those interested in using biological databases, we present the history of these databases, their benefits for modern science, and the challenges inherent to their specific content. We also outline the scientific and policy context surrounding COP16, the United Nations Conference on Biological Diversity in 2024 [36].

The increasing availability of commercial genome sequencing for the general public, driven by increasingly affordable testing costs, may pose ethical and data security risks. Selected artificial intelligence (AI) models have been used for some time to screen job candidates [33]. This screening process is highly discriminatory, raising questions about whether AI could unconsciously discriminate and make selections based on a selected genotype. The results of polygenic risk assessments and knowledge of individual variants strongly associated with health outcomes raise questions about the potential use of such data in the insurance process for individuals, families, and whole populations. The need to treat individuals’ genomic sequences as personal data has been recognized in the European Union and beyond, which has implications for data storage and processing policies [25,33,34,36].

It is recognized that growing data resources require coordination and financial support necessary to maintain databases and ensure open access to data. The Global Biodata Coalition [37] aims to bring together global research funders to share knowledge and strategies for supporting biodata resources while also facilitating the development of principles and models for the coordinated funding of key global biodata resources. The inclusion of the database in the Global Biodata Coalition (GBC, https://globalbiodata.org/ (accessed on 29 April 2026)) is intended to facilitate coordination of database funding and open access to biodata resources for all researchers worldwide for both scientific and commercial purposes [37].

Johns Hopkins University presented, in their guide, primarily genomic data resources available on the National Center for Biotechnology Information platform, with most of the data being openly accessible from government and non-commercial sources. Data on genome-wide association studies (GWASs); single-nucleotide polymorphism (SNP) arrays; genome sequencing; and transcriptomic, epigenomic, and gene expression data are available [38].

Another website gathering information on global biological databases, Database Commons (https://ngdc.cncb.ac.cn/databasecommons/ (accessed on 29 April 2026)), is maintained by the National Genomics Data Center of the China National Center for Bioinformation/Beijing Institute of Genomics, Chinese Academy of Sciences [39]. The authors have compiled a list of databases sorted by categories: (1) raw biological data; (2) gene, genome, and annotation; (3) genotype, phenotype, and variation; (4) phylogeny and homology; (5) expression; (6) modification; (7) structure; (8) interaction; (9) pathway; (10) health and medicine; (11) standard, ontology, and nomenclature; (12) metadata; and (13) literature references [40,41].

Authorized access to selected databases is required, such as the UK Biobank [42], Genomics England [43], and the European Genome-phenome Archive, EGA [44]. Other databases require subscriptions and fees, such as the Eukaryotic Pathogen, Vector, and Host Informatics Resources [45] or the BioCyc databases [46]. Many private databases offer genome sequencing and analyses of genomic data downloaded from external sources, and some companies offer basic analyses free of charge after creating an account. Of course, the security of genomic data depends on the company. For example, Dante Labs, New York City, United States [47] states that it does not sell personal or genomic data, and customers can enter, delete, or anonymize their data at any time, in accordance with the USA Health Insurance Portability and Accountability Act (HIPAA) and European General Data Protection Regulation GDPR guidelines. Similarly, Genomelink, Berkeley, United States [48] offers free testing of selected traits and ancestry, including data obtained from other companies, such as 23andMe, AncestryDNA, and MyHeritage. Other genome testing companies include Nebula Genomics, San Francisco, United States [49], which states that it does not sell customer data and that participation in scientific research requires consent. Similar restrictions apply to the Living DNA, Frome, England [50]. FamilyTreeDNA, Houston, United States [51] allows the use of customer data for scientific research and access to genomic data by law enforcement agencies after the customer has provided consent.

Circle DNA Company, Hong Kong, China [52] uses a next-generation sequencing approach for analysis and claims it does not share or sell data for research purposes. SelfDecode, Miami, United States [53] indicates that it never sells data without the customer’s express consent, that genetic data are anonymized, and that they are not shared with employers, insurance companies, or any other external entities. Sequencing, Pasadena, United States [54] provides whole-genome sequencing and does not share or sell data to third parties.

23andMe, South San Francisco, United States [55] states that it requires explicit consent to use data. Data may be shared with designated individuals, including family and physicians. In the event of a bankruptcy, merger, acquisition, reorganization, or sale of assets, personal data may be shared, sold, or transferred as part of that transaction. 23andMe will not share information with law enforcement unless required by law to comply with a valid court order, subpoena, or search warrant.

On the Ancestry platform, Lehi, United States [56], data is shared for marketing purposes with explicit consent, similarly to the use of data for research purposes by non-profit organizations and commercial companies. A court ruling justifies sharing data with law enforcement authorities. MyHeritage, Or Yehuda, Israel [57] never sells or licenses personal information, including genetic or health data, to insurance companies, government agencies, or other companies or employers. You can withdraw your consent at any time.

## 8. The Understanding of the Human Genome Has Enabled the Study of Related Species

An example of comparative studies of humans with extinct members of the genus Homo is the Neanderthal genome. In 2008, Svante Pääbo’s team sequenced the entire Neanderthal mitochondrial genome from the bones of an individual who lived 38,000 years ago [58]. They observed that Neanderthals had a significantly higher proportion of nonsynonymous mutations compared to synonymous mutations in the protein-coding mitochondrial gene sequences. Furthermore, the Neanderthal mitochondrial lineage was 20% shorter than that of humans, three times shorter than would be expected based on the side effect of the sample’s age. This difference was attributed to the random occurrence of substitutions.

In 2010, the first draft of a Neanderthal nuclear genome was sequenced, using the samples from Vindija Cave in Croatia, altogether comprising 21 ancient bones [59]. It was achieved with highly robust scientific methods and a great dose of adaptability. The average divergence of anatomically modern humans and Neanderthal DNA sequences was estimated to have occurred 825,000 years ago, while the populations separated from each other 270,000 to 440,000 years ago. Additionally, the sequenced Neanderthal genome was used to detect genes potentially subjected to positive selection early in the history of our species [58,59,60].

The field of paleogenetics has witnessed significant changes. Initially focused on the analysis of short DNA sequences, it has come a long way, resulting in the analysis of entire genomes and the conduct of metagenomic studies. Despite initial challenges in validating results, paleogenetics is a newly established field. Significant improvements in genomic research methodology have been achieved through the incorporation of next-generation sequencing (NGS) technology, the development of protocols for isolating highly fragmented DNA, and the establishment of standards for result validation [59,60]. As NGS technologies continue to develop and become increasingly affordable, the scientific community will continue to analyze ancient genomic data to analyze how individuals and human populations migrated throughout the genetic history of the regions studied. As we advance, because genomic data reveal a partial picture, researchers will likely also focus on multi-disciplinary studies. These analyses will also draw on paleoproteomics, paleogenetics, metagenomics, and, potentially, paleoepigenetics [60]. With the further development of NGS technologies and their increasing affordability, the scientific community will continue to analyze ancient genomic data to achieve the finest possible resolution of the genetic history of our world. Additionally, as genomic data alone rarely provide a complete picture, scientists in the future should likely focus on multi-disciplinary studies integrating paleogenetics, paleoproteomics, meta-genomics, and possibly paleoepigenetics [61].

## 9. Legal Considerations

The legal framework for DNA testing worldwide varies greatly and depends on the purpose of the test (medical, forensic, or genealogical) and the jurisdiction of the given country. Regulations range from complete bans on private testing (e.g., France) to a liberal market approach (e.g., the USA), with a strong emphasis on the protection of genetic data (General Data Protection Regulation (GDPR) in the EU). The use of genetic testing is not limited to scientific, medical, or forensic purposes; data derived from genome sequencing analysis is also used by the pharmaceutical industry, which must base its research on the analysis of large, population-based DNA biobanks.

The main areas of the legal framework for DNA testing involve five dimensions. The first area encompasses paternity and relationship testing. For the result to be legally valid, all individuals undergoing testing (especially the child and the putative father) must provide informed consent. In many countries, a court cannot physically compel an individual to provide a DNA sample. However, refusing to perform a court-ordered test may result in financial penalties or negative legal consequences (the court may recognize paternity). The European Court of Human Rights (ECtHR) has ruled that a court order for DNA testing in paternity cases does not violate the right to private life. A vast trove of genomic data resides in the hands of police and other agencies. The Federal Bureau of Investigation’s (FBI) National DNA Index (NDIS) contains over 19,272,496 criminal profiles; 6,142,751 arrest profiles; and 1,449,731 forensic profiles (as of November 2025). Ultimately, the success of the CODIS (Combined DNA Index System) program will be measured by the number of crimes that it helps solve [62]. The primary CODIS metric, “Investigative Assistance,” tracks the number of criminal investigations in which CODIS added value to the investigative process. As of November 2025, CODIS had generated over 781,492 hits, assisting in over 758,449 investigations. Offender profiles in the NDIS include profiles of convicted, detained, and legal persons. Central Forensic Laboratories of the Police collect, store, and process genetic data in the DNA Databases. The DNA database may contain genetic profiles of individuals suspected of committing crimes prosecuted at the public prosecutor’s office, minors committing prohibited acts, individuals whose identity is unknown or who are attempting to conceal it, and wanted individuals. The database receives profiles of unidentified corpses and unidentified traces from crime scenes and other procedural activities.

The second area involves direct-to-consumer (DTC) genetic testing [63,64]. In Europe, this approach is very restrictive. France prohibits commercial DNA tests (ancestry and health) conducted by private companies. In other countries, such as Germany and Switzerland, health tests must be conducted under medical supervision. In the USA, the market is open, and companies such as 23andMe offer tests without the need for a doctor, although the FDA (Food and Drug Administration) oversees their accuracy. Since May 2022, DTC diagnostic tests in the EU have been subject to the In Vitro Diagnostic Medical Devices Regulation (IVDR), which limits their commercial freedom in health matters. DNA sequence analysis has become affordable for a large number of patients. The cost of testing all protein-coding genome fragments, known as whole-exome sequencing (WES), is approximately 1000 USD, with significantly cheaper options also emerging, offering the test at promotional prices of only a few dozen dollars. Analysis times were dramatically accelerated, reducing the test to approximately 5 h, which is particularly important in emergency medicine. Sequence analysis requires small amounts of biological material, primarily epithelial cells collected from the inside of the cheek (a cheek swab), although blood is still preferentially used as the starting material. Obtaining high-quality materials for further testing is crucial. Non-standard materials, such as all types of biological traces (hair, traces of secretions or excretions, clothing, fingerprints on everyday objects, etc.) widely used in forensic medicine, can also be a source of information.

The third area refers to data protection and genetic privacy [63,64]. In the European Union, genetic data are considered particularly sensitive (GDPR). Its processing requires explicit consent, and companies must guarantee a high level of security. In the USA, the Genetic Information Nondiscrimination Act (GINA) protects against discrimination by health insurers and employers based on DNA test results. In many countries (e.g., the UK, Australia), regulations exist that prohibit insurers from using DNA test results to increase premiums or deny coverage [65,66,67]. Genetic information is certainly of interest to insurance companies and employers, who can thus estimate the level of risk associated with insurance premiums and the ability to work efficiently. Not for the first time in the history of science, technical capabilities are outpacing the adaptation of legal regulations and ethical standards. In many countries, genetic genealogy testing is becoming increasingly popular and readily available. This type of testing was initiated and has been performed primarily in America for years. Companies offering such services include the American companies AncestryDNA, 23andMe, and FamilyTreeDNA, as well as the Israeli company MyHeritage. All companies compare the information obtained with previous data.

The fourth area focuses on forensic research. Police in most countries (e.g., the US, UK, Poland) have the right to collect DNA samples from individuals suspected or convicted of serious crimes and store them in databases. The growing popularity of private databases (e.g., GEDmatch) has led law enforcement agencies (e.g., in the US) to use them to identify criminals, which raises ethical concerns but is permissible in criminal investigations.

The fifth area refers to genetic counseling. In many jurisdictions (e.g., Austria, Germany, Portugal, Spain), the law requires that health-related genetic testing be preceded and followed by a consultation with a geneticist to ensure that the patient understands the implications of the results.

The substantial importance of genetic data protection is also evidenced by Russia’s recent adoption of a law ensuring the security of such data. The Russian Federation’s latest Federal Law No. 43-FZ of 20 February 2026 introduces significant changes to regulations in the field of genetic engineering and to the protection of citizens’ genetic data [68]. The main objective of the amendment is to strengthen national security and sovereignty in the collection, storage, and analysis of human genetic data. Among other things, these regulations prohibit the transfer of the genetic data of Russians obtained in population studies to foreign entities without special permits (with exceptions, e.g., for the treatment of a specific patient). The new provisions define the legal basis for the transfer of data and introduce precise definitions of biomedical concepts into the legal system. Human genetic data are defined as information of the sequence of nucleotides in nucleic acid polymers and their chemical modifications obtained as a result of the molecular genetic testing of biological material. Population studies are defined as genetic or immunological studies conducted on groups of people (more than three people) who share a common characteristic, such as age, gender, nationality, place of residence, or health status. The changes in the regulations are aimed at protecting and controlling the transfer of genetic information, including limiting its uncontrolled flow (e.g., abroad). An obligation to ensure the security and protection of genetic data has been introduced, including strict rules on the transfer of genetic data—such as data obtained during population or immunological studies—to foreign natural and legal persons (both in Russia and abroad) and foreign states. The transfer of data includes, among other things, the collection, storage, sharing, transmission, dissemination, and placement of data on telecommunications networks (e.g., the Internet). Legal entities, officials, citizens of the Russian Federation, and foreigners are liable for violating the new provisions on the transfer of genetic data. The act is considered to be part of broader efforts to limit the uncontrolled outflow of Russians’ genetic data abroad. The transfer of data outside the Russian Federation is only permitted in exceptional circumstances, e.g., for the purpose of providing medical assistance to a specific patient, manufacturing medicines, or international cooperation, subject to certain conditions. The act is due to enter into force on 1 September 2026.

The European Union’s General Data Protection Regulation (GDPR) protects personally identifiable information, including the collection, processing, storage, and transfer of personal data of citizens or residents of the European Union or European Economic Area [69]. It is assumed that the regulations are the most restrictive and also enforce protection outside the EU. Special or sensitive data also include genetic and biometric data, as well as any health records. Recital 34 defines genetic data as personal data relating to hereditary or acquired genetic characteristics obtained through the analysis of biological samples, such as DNA or RNA. Such data may reveal unique and sensitive information regarding a person’s identity and health. This recital is important because it emphasizes that genetic data is highly sensitive and requires stronger protection under the GDPR, ensuring that organizations process such data under strict safeguards and in accordance with lawful conditions. GDPR considers international data transfers as all the transfers of personal data to or from a country outside the European Economic Area (EEA). Any international transfer of personal data outside the European Economic Area may only be carried out in full GDPR compliance.

Under European Union law, the processing of health and genetic data—which constitutes special categories of personal data within the meaning of the General Data Protection Regulation (GDPR)—is prohibited under Article 9(1) of the GDPR, unless at least one of the ten exceptions set out in Article 9(2) is met. The practical application of the principles for research purposes and in relation to the possibility of introducing new, personalized therapies is a significant challenge [65,70]. An individual’s genetic data may only be processed with their consent, which must be freely given, specific, informed, and unambiguous. Consent may be withdrawn at any time (Article 7(3)), in which case the controller must cease processing and erase the data upon request. Furthermore, the data may only be used for the specific purposes for which consent was given, with separate consent required for each new purpose. The GDPR provides for a wide range of rights related to genetic data, including the following: (1) the right of access (Article 15) to a full copy of all stored genetic data, along with information on how it is processed and to whom it has been disclosed; (2) the right to erasure (Article 17); (3) the right to data portability (Article 20); (4) the right to restriction of processing (Article 18); (5) the right to object (Article 21) to certain types of processing, including profiling. Non-compliance carries severe financial penalties.

In the United Kingdom, genetic/genomic data protection is recognized under the UK General Data Protection Regulation (UK GDPR, Regulation (EU) 2016/679 of the European Parliament and of the Council of 27 April 2016 on the protection of natural persons with regard to the processing of personal data and on the free movement of such data (United Kingdom General Data Protection Regulation)) [71] and the Data Protection Act 2018 [72].

In the Data Protection Act 2018, protection applies to genetic data belonging to special categories of personal data, i.e., personal data relating to the inherited or acquired genetic characteristics of a natural person that provide unique information about the physiology or the health of that natural person and result, in particular, from an analysis of a biological sample from the natural person in question.

In the United States, genetic data are protected under the Health Insurance Portability and Accountability Act (HIPAA) [73] and the Genetic Information Nondiscrimination Act (GINA) [74], providing limited protections. However, a substantial problem is the lack of solutions enabling data protection during ownership changes within companies [75]. GINA prevents discrimination on the basis of genetic characteristics by health insurers and employers, but it does not cover data processing, data storage, or obligations relating to data breaches. The HIPAA regulations apply only to healthcare providers, insurers, and their business associates. Consumer genomics companies that sell directly to consumers are not usually subject to this legislation.

Some states, such as Illinois, California, Montana, Texas, and Florida, have enacted laws protecting the privacy of genetic data; however, most states lack specific laws regulating the protection of genetic data. On 22 May 2025, a bipartisan group of senators introduced the “Don’t Sell My DNA Act,” a bill that aims to amend the federal bankruptcy code by, among other things, requiring notice and explicit consumer consent before the use, sale, or lease of genetic information in the context of bankruptcy proceedings [76]. Some regions of Asia are actively developing large-scale national databases and employing data-sharing models that differ from those used in the EU; for example, China restricts the transfer of data across national borders. Japan, on the other hand, has adopted measures similar to those used in the EU regarding data protection in the private sector.

In 2024, the World Health Organization (WHO) published the Guidelines for the Collection, Access, Use, and Sharing of Human Genome Data. These guidelines provide a global approach to protecting individual rights, promoting equality, and fostering responsible collaboration in genomic research [63].

It is believed that the current regulations regarding the protection of genetic data are clearly insufficient globally. The recent bankruptcy of 23andMe highlights the risks associated with the sale and potential misuse of genetic data and biological samples [64]. The solution is not to explicitly prohibit the commercialization and sale of unique, immutable, and deeply personal data derived from DNA analysis but rather to ensure data privacy and protection against discrimination at a global level [31,32]. Oversight of the genetic testing sector and scientific institutions conducting genomic research projects is also necessary. Otherwise, the sale of 23andMe could set a dangerous precedent—treating DNA as a mere corporate asset could undermine public trust in genetic testing and the protection of particularly sensitive data.

In summary, there is a global trend toward the stricter protection of genetic data, accompanied by a boom in commercial genealogy testing, which often operates in a “gray zone” of privacy.

## 10. Genomic Data Protection: Threats and Technology Solutions

Genomic data available in public databases require privacy and security. Machine learning methods are being used to secure data while maintaining data confidentiality, such as differential privacy, federated learning, and synthetic data generation using generative adversarial networks (GANs) [77]. Storing genomic data in external cloud services increases the vulnerability of genomic data to cyber threats and unauthorized access, enabling re-identification, membership inference, and link-based attacks.

There is a wide range of attacks on genomic data. Identity-tracking attacks use anonymized genomic data along with quasi-identifiers (e.g., age, gender, name) to re-identify individuals. Inference attacks rely on partial information to infer additional related data, potentially revealing sensitive genomic details. Supplementary attacks use publicly available DNA databases or family member DNA sequences to reconstruct all or part of an individual’s genetic information. Bayesian networks in genetic attacks rely on probabilistic graphical models to infer missing genomic data and relationships between genetic markers. Machine-learning-based re-identification uses artificial intelligence models trained on genomic sequences to match anonymized data to identified datasets, rendering anonymization attempts more difficult. Adversarial attacks on genomic AI models involve the deliberate manipulation of genomic AI models through disruption, which may lead to misclassification or the extraction of sensitive genomic data [77].

Several solutions are currently used to protect against attacks on genomic data. Data perturbation methods via differential privacy (DP) add noise to genomic data before sharing or analysis to protect user privacy [78]. Another solution involves K-anonymity data aggregation, which generalizes or groups genomic attributes to prevent unique identification. Machine-learning-based solutions can use differentially private stochastic gradient descent (DP-SGD), based on differentially private training methods, to prevent data leakage in machine learning models. Federated learning (FL), on the other hand, enables decentralized model training on genomic data without the need to share raw data. Cryptographic solutions rely on homomorphic encryption (HE), which allows computations to be performed on encrypted genomic data without the need to decrypt it [79]. Another approach, related to secure multiparty computation (SMC), enables collaborative genomic analysis while maintaining the privacy of individual data. Blockchain-based security for genomic data sharing utilizes distributed ledger technology to control access and maintain immutable records of genomic data transactions [80,81]. Another approach relies on generating synthetic data using generative adversarial networks (GANs)—synthetic data models within a deep learning framework—to generate synthetic genomic datasets with guaranteed privacy. Access control solutions are implemented at the permission level, restricting access to genomic data based on institutional approvals (e.g., NIH-controlled access databases) or at the participant level and allowing users to control who has access to their genomic data. Another approach involves logging trusted but verified data, ensuring auditing and tracking access to genomic data through immutable logs [77].

The tensions between individual rights and public health have been discussed for many years, and they concern not only genomic data but also infectious diseases, chronic diseases, and the right of an individual to, for example, ride a motorcycle without a helmet, which may generate significant involvement of public services [66,67]. Access to genomic data is essential for advancing knowledge, understanding genetic diversity, and supporting international public health [82].

The need to ensure genomic privacy impacts the need to ensure an appropriate balance between individual safety and public safety, as well as the ability to use genomic information collected in databases to ensure health and safety at the population level— for example, in the context of the recent COVID-19 pandemic or in preparing to address emerging health challenges globally [83,84]. Collective security, for example, regarding infectious diseases combined with the possibility of targeted biological attacks, must balance the needs of the community with ensuring the privacy of genomic data. Ethical, legal, and technological challenges (including federated learning, differential privacy, and homomorphic encryption) arise regarding surveillance; genomic privacy protection; and the collection, use, and sharing of genomic data. Building knowledge and understanding of the biosecurity of genomic data in society remains equally important. International cooperation is, of course, essential, along with ensuring equivalent security systems for managing emerging health threats [85]. Does defining the boundaries between “necessary” and “proportionate” genomic privacy restrictions for biosecurity surveillance require a solution? Identifying the effectiveness of technologies protecting genomic data and monitoring mechanisms that enable the development of public trust and protect against abuse is crucial. Ensuring the protection of genomic data is crucial, with particular attention focused on vulnerable groups. Additionally, international cooperation free from political influence and national interests remains a significant challenge [86,87].

The need for new solutions, particularly in the area of infectious diseases, with particular emphasis on COVID-19, is evidenced by the WHO’s global genomic surveillance strategy for pathogens with pandemic and epidemic potential [88].

## 11. Conclusions

If we continue to carelessly share patient data for high-throughput testing, we are not only building databases for commercial entities but also, instead of developing our own science, dumping genetic data into a completely unidentified online space, where it can be used for unethical or even bioterrorist purposes. We urgently need a global strategy for genomic data protection based on awareness, control, and accountability.

The legal framework for DNA testing worldwide varies greatly and depends on the purpose of the test (medical, forensic, or genealogical) and the jurisdiction of the given country. The use of genetic testing is not limited to scientific, medical, or forensic purposes; data derived from genome sequencing analysis is also used by the pharmaceutical industry, which must base its research on the analysis of large, population-based DNA biobanks.

According to WHO guidelines, genomic data collection should be based on informed consent and privacy, promoting equality between diverse populations for research benefits. Collaboration between governments, academia, and private entities should foster responsible data sharing, supported by robust governance structures, to promote global health while respecting privacy [89]. Capacity building in regions with limited genomic infrastructure will be important. We must maintain a balance between public health and privacy, particularly in terms of protection against pathogens, cancer, and the risks associated with processing, storing, and sharing genomic data—including epigenetics—and review current privacy protection strategies.

## Figures and Tables

**Table 1:** Examples of major types and formats of genome-related data.

Format	Description	File
FASTA (.fasta, .fa, or .fna) Plain Sequences, 1985	Storage of nucleotide sequences or amino acid sequences. Raw data.	Single-line header preceded by the “>“ symbol. The sequence is stored on subsequent lines (80 characters/line). No additional information is provided. the basis of queries in the infamous BLAST + 2.17.0 server (NCBI)
FASTQ (.fastq or .fq) Sequences with Quality Scores, 2000	Storage of nucleotide sequences or amino acid sequences and their quality scores.	Four lines. The sequence identifier with an optional description (starting with a “@” symbol), the raw sequence, a separator line (usually beginning with a “+” symbol), and the sequence quality scores. Quality scores are encoded as ASCII characters, each representing the probability of a sequencing error for a given base in the PHRED output. Output from sequencers is typically saved in FASTQ format.
SAM/BAM/CRAM (.sam/.bam/.cram) Alignments	Storage of sequence alignment information and differences between the sample and the baseline genome: Sequence Alignment/Map SAM, Binary Alignment/Map BAM, and Compression Alignment/Map CRAM.	SAM is a tab-delimited, human-readable text format containing alignment information and additional metadata. BAM’s compressed binary form is the binary equivalent of SAM; fast processing and reduces memory requirements. CRAM compresses alignment information by storing only the differences between aligned sequences and the reference sequence. This significantly reduces storage space requirements but requires access to the reference sequence.
BED/GTF (.bed/.gtf) Genomic Annotations	Storage of gene and feature annotations: Browser Extensible Data BED and Gene Transfer Format GTF.	A BED file is a tab-delimited text file that defines rows of data representing a distinct feature (e.g., a gene or transcript, epigenetic markers) with fields for chromosomal coordinates and additional annotations. The GTF format offers a more structured format and additional fields for storing data related to genomic features (e.g., exons, genes, transcripts, their corresponding locations).
Bedgraph Coverage Data	Storage of continuous data, such as gene expression levels or genome coverage. Efficient representation of large numerical datasets.	Bedgraph is a tab-delimited text file (similar to the BED format), in which each line defines a chromosome region (e.g., chromosome, start, end) and its associated continuous value.
VCF/GFF (.vcf/.gff) Functional analysis	Storage of genetic variations in comparison with reference genome: Variant Call Format VCF and general feature formats GFF. Functional analysis.	Variant Call Format files are used to store genetic variants (SNPs and indels). These files are small and easy to manage. Generic feature formats (GFFs) are much larger and contain more detailed information about the sequence and the features within that sequence (sequence name, type of feature described, e.g., entire gene, exon, or intron; coordinates of the feature within the entire sequence; and other information such as the parent gene on which the exon is located).
Loom file, a type of Hierarchal Data File (HDF5)	Storage of large amounts of omics data and metadata of one cell.	Loom files contain a main matrix, optional additional layers, a variable number of row and column annotations, and sparse graph objects. Under the hood, Loom files are HDF5 and can be opened from many programming languages, including Python, R, C, C++, Java, MATLAB, Mathematica, and Julia. Human Cell Atlas.
Mascot Generic Format MGF (.mgf)	Storage of mass spectrometry fragmentation data for peptide identification.	Used in proteomics and metabolomics (mass, charge, and abundance) for efficient computational analysis of data.
MZXML (.XML)	Storage of mass spectrometry data.	An open data format for storage and exchange of mass spectroscopy data. mzXML provides a standard container for ms and ms/ms proteomics data.
Protein Data Bank PDB (.pdb)	Storage of atomic coordinates from protein sequences.	Data deposited in the Protein Data Bank at the Research Collaboratory for Structural Bioinformatics. They can be used alongside software such as Pymol to predict protein structures and assess the impact of mutations, their relationship with other proteins, and much more.
Wiggle/BigWig (.wig/.bw)	Storage of genome-wide signal data: wiggle file, BigWig files.	Wiggi file use “bins” (DNA methylation, GC percentage or histone modification levels). BigWig, the binary equivalent. The data from a wiggle file can be plotted using specialized software, allowing for visualization of biological features.

## Data Availability

Data sharing is not applicable. No new data were created or analyzed in this study.
